# Outcomes of single-stage total arch replacement via clamshell incision

**DOI:** 10.1186/1749-8090-6-114

**Published:** 2011-09-20

**Authors:** Hiroto Iwasaki, Hisashi Satoh, Toru Ishizaka, Hikaru Matsuda

**Affiliations:** 1 Department of Cardiovascular Surgery, Higashi Takarazuka Satoh Hospital, Nagao-cho 2-1 Takarazuka, Hyogo 665-0873, Japan

**Keywords:** total arch replacement, clamshell approach, arch-first technique

## Abstract

**Background:**

Treatment of complex aortic pathologies involving the transverse arch with extensive involvement of the descending aorta remains a surgical challenge. Since clamshell incision provides superior exposure of the entire thoracic aorta, we evaluated the use of this technique for single-stage total arch replacement by arch vessel reconstruction.

**Methods:**

The arch-first technique combined with clamshell incision was used in 38 cases of aneurysm and aortic disease in 2008 and 2009. Extensive total arch replacement was used with clamshell incision for reconstruction of arch vessels under deep hypothermic circulatory arrest.

**Results:**

Overall 30-day mortality was 13%. The mean operating time was approximately 8 hours. Deep hypothermia resulted in mean CPB time exceeding 4.5 hours and mean duration of circulatory arrest was 25 minutes. The overall postoperative temporary and permanent neurologic dysfunction rates were 3% and 3% for elective and 3% and 0% for emergency surgery, respectively. All patients except the five who died in hospital were discharged without nursing care after an average post-operative hospital stay of 35 days.

**Conclusions:**

The arch-first technique, combined with clamshell incision, provides expeditious replacement of the thoracic aorta with an acceptable duration of hypothermic circulatory arrest and minimizes the risk of retrograde atheroembolism by using antegrade perfusion.

## Introduction

The treatment of complex aortic pathologies involving the transverse arch with extensive involvement of the descending aorta remains a surgical challenge because distal anastomosis is often impossible through conventional median sternotomy [1-3]. Several techniques have been used to overcome this challenge and these include: additional thoracotomy, the elephant trunk technique (both classical and frozen), the pull-through technique with posterior pericardiotomy, and staged repair [4-6]. Another option is proximal anastomosis with posterolateral thoracotomy, which is a popular surgical approach for Stanford type B aortic dissection [7]. However, no optimal surgical technique or approach for the treatment of such lesions has been established. Kouchoukos et al. recently reported that the single-staged, arch-first replacement technique with a clamshell incision is a safe and effective procedure for patients who require extensive reoperations for chronic expanding type A dissection [8]. However, small studies such as this are not ideal for precisely evaluating these methods, and only one similar study has been published [8]. Thus, large-scale similar studies would be required in the future. We used a single-staged, arch-first replacement technique with a clamshell incision in 38 patients to treat extended aortic arch disease involving the descending aorta.

## Materials and Methods

### Patients

The modified arch-first technique has been used for patients with arch or distal arch disease (aneurysm or dissection) involving the left subclavian artery extending as far as the descending aorta. Cases of aortic arch disease involving the descending aorta replaced through a left thoracotomy have been excluded from this study.

In 2008 and 2009 the modified arch-first technique was used by our institution by two surgeons for 38 consecutive cases, comprising 20 males and 18 females with an average age of 71 years (range: 42 - 93 years). There were 12 true aneurysms, 6 ruptured aortic aneurysms, 5 acute and 8 chronic type B aortic dissections and 7 acute type A aortic dissections, all with distal arch aortic disease. Eighteen cases underwent emergency surgery. Concomitant procedures consisted of aorto-coronary bypass grafting, Bentall operation, aortic valve replacement and aorto-femoral bypass grafting. There were 10 cases of repeat surgery, with the previous surgery consisting of three ascending aortic replacements, one aortic valve procedure, one coronary artery bypass and five cases of abdominal aortic repair. In all cases, the arch vessels were reconstructed with a commercially available four-branched graft (Hemashield; Boston Scientific, Natick, MA).

### Operative procedure for the arch-first technique

Following the insertion of monitoring catheters, the induction of anesthesia and the placement of a double lumen endotracheal tube, the patient is placed in the supine position with the left arm elevated to the head at a 160° angle, and the chest entered through a clamshell incision (bilateral 4^th ^intercostal incision and transverse sternotomy). Cardiopulmonary bypass (CPB) is established by using the right femoral vein for venous drainage and the ascending aorta or femoral artery for arterial return, followed by the initiation of cooling. The left heart is vented with a catheter inserted into the right upper pulmonary vein, and a cannula inserted into the coronary sinus for delivery of a cold blood cardioplegic solution for myocardial protection. The head is packed in ice, and methylprednisolone (7-10 mg/kg) and thiopental (10-15 mg/kg) administered intravenously during cooling. When the bladder temperature reaches 20°C, the patient is placed in the Tredelenburg position and deep hypothermic circulatory arrest established. Retrograde cardioplegia is used as soon as the ascending aorta is opened without cross clamping following circulatory arrest and every 20 minutes thereafter. While cooling is being effected, a long, collagen-impregnated woven Dacron aortic graft is selected and prepared for anastomosis. When coronary bypass grafts are required, distal anastomosis is performed during cooling. Aortic valve replacement or reconstruction of the aortic root, if indicated, is also performed at this time. After the left subclavian artery has been clamped in the left pleural space, the ascending aorta and aortic arch are opened while taking care to avoid dislodgment of atheromatous debris. The brachiocephalic arteries and left common carotid arteries are transected at their orifices, and each vessel reconstructed with a four-branched arch graft with a 4-0 polypropylene running suture; first the left common carotid artery and then the brachiocephalic artery. The blood temperature is kept at approximately 18-20°C. Following completion of the two arch vessel reconstructions, antegrade cerebral perfusion is resumed through the side branch of the aortic graft, and air is evacuated from the proximal open end of the aortic graft. The arch graft is then clamped at both ends and at the third branch, followed by the establishment of antegrade flow through the two arteries. The perfusion flow is maintained at 10-15 mL/kg/min so that the right radial artery pressure remains above 30 mmHg. The most distal branch of the aortic graft is anastomosed to the left subclavian artery in the left pleural space and antegrade flow is established through the three arteries while maintaining the same flow rate, pressure and temperature. The posterior wall of the arch aneurysm is then incised to the descending aorta, while the anterior wall of the arch is never dissected to avoid injury to the recurrent laryngeal and phrenic nerves. Next, the prepared graft is passed down through the opening under the pedicle containing the vagus and phrenic nerves and is cut to the appropriate length. Distal anastomosis is performed with an open technique and the same graft is used with a 4-0 polypropylene running suture and Teflon felt strip reinforced on the adventitia of the aorta. After completion of the distal anastomosis, debris and air are washed out from the descending aorta by perfusing blood from the femoral artery cannula inserted before the start of CPB. The distal arch clamp is then removed and antegrade systemic perfusion is reinitiated through the side branch of the arch graft followed by complete rewarming. Finally, the ascending aorta is transected and proximal anastomosis is established with a 3-0 polypropylene running suture under perfusion from the side branch of the graft, and reconstruction of the aortic arch is completed. The saphenous vein bypass grafts are anastomosed to the aortic graft at this time and routine maneuvers to de-air the heart are performed. A needle vent is then placed on the proximal portion of the graft and connected to suction, after which the clamp proximal to the arch is removed. Hemostasis is effected and the patient is weaned from CPB. Finally, the 10 mm graft is divided, the suture is ligated close to the aortic graft and four thoracostomy tubes are placed in each pleural space.

### Statistical Analyses

Data are expressed as mean ± standard deviation or percentage. Intergroup comparisons were carried out with Fisher's exact test, χ^2 ^tests and t-tests as appropriate. A *p *value < 0.05 was considered significant. The results were statistically analyzed using a software package (Statview 5.0, Abacus Concepts Inc., Berkeley, CA).

## Results

The technique described in the previous section was used for 38 patients undergoing total arch replacement (Table 1). The overall 30-day mortality was 13% (5 patients). One of the 20 patients (5%) undergoing elective surgery died during the first 30 postoperative days, as did 4 of the 18 patients (22%) undergoing emergency surgery. Four of the latter patients showed rupture of an aortic aneurysm. Of the patients treated with elective surgery, one died postoperatively of multiple organ failure due to shaggy aorta syndrome which developed during repair of an aneurysm. Three patients died from low output syndrome after emergency repair of a ruptured aneurysm and aortic dissection, and another died from acute myocardial infarction after emergency repair of acute aortic dissection associated with a distal arch aneurysm. Three additional patients died during hospital stay from complications of myocardial infarction, stroke and pneumonia 86, 335 and 35 days, respectively, after emergency surgery. The 6-month mortality rate was 21%.

**Table 1:** Preoperative patient characteristics

Patients	No. of patients
Male/female ratio	26:12
Age (y)	71 ± 10
Emergency cases	18 (47%)
Comorbidity	
Hypertension	32 (84%)
History of smoking	17 (45%)
Hyperlipidemia	8 (21%)
Chronic obstructive pulmonary disease	7 (18%)
Coronary artery disease	3 (8%)
Peripheral vascular disease	0 (0%)
Creatinine ≥ 1.5 mg/dL	3 (8%)
Previous transient ischemic attack or stroke	2 (5%)
Marfan syndrome	1 (3%)
Diabetes mellitus	2 (5%)
Previous operations	
Coronary artery bypass	1 (3%)
Aortic valve procedure	1 (3%)
Ascending aortic repair	3 (8%)
Descending thoracic aortic repair	0 (0%)
Abdominal aortic aneurysm repair	5 (13%)
Etiology of aortic disease	
Degenerative aneurysm	12 (32%)
Ruptured aneurysm	6 (16%)
Acute type A dissection	7 (18%)
Acute type B dissection	5 (13%)
Chronic type B dissection	8 (21%)

Perfusion data are summarized in Table 2. The mean operation time was approximately 8 hours, and the mean CPB time exceeded 4.5 hours as a result of the use of deep hypothermia with an average minimum bladder temperature of 20°C. The mean duration of circulatory arrest was 25 minutes and selective cerebral perfusion through the side branch of the arch graft lasted 73 minutes. Lower body ischemic time was 97 minutes because distal anastomosis was performed, while cardiac ischemic time was nearly 2.5 hours, during which the entire aortic arch reconstruction procedure was performed.

**Table 2:** Clinical outcomes

	Mean ± Standard deviation
Operation time (minutes)	472 ± 109
Total CPB time (minutes)	269 ± 72
Myocardial ischemia time (minutes)	141 ± 41
Circulatory arrest time (minutes)	25 ± 5
Selective arch perfusion time (minutes)	73 ± 27
Lower body ischemia time (minutes)	97 ± 28
Cooling time (minutes)	48 ± 10
Rewarming time (minutes)	64 ± 14
Bleeding (mL)	827 ± 376
Transfusion (mL)	1249 ± 671
ICU stay (days)	9.2 ± 8.9
Intubation time (days)	6.6 ± 8.2
Postoperative hospital stay (days)	35.0 ± 27.1

All patients were weaned from mechanical ventilation. While 10 patients were extubated within 24 hours, the average intubation period was nearly 6 days. Four patients (10.5%) with preexisting respiratory insufficiency were ventilated for more than 48 hours and two of them required temporary tracheostomy. The mean intensive care unit stay was 9 days.

The overall postoperative temporary and permanent neurologic dysfunction rates were, respectively, 3% and 3% for elective and 3% and 0% for emergency surgery. Temporary psychiatric disorders, such as delirium and convulsion, were observed in two cases (6%). Other morbidities were pneumonia (4 cases), myocardial infarction, low output syndrome requiring high dose inotropic support, and renal dysfunction requiring temporary dialysis to attain a serum creatinine level of over 3.0 mg/dl. One patient did not awaken, and four patients suffered from delayed awakening. There were no significant differences between elective and emergency surgery in terms of the awakening or intubation period, but emergency surgery involved a significantly longer stay in ICU and in hospital after surgery. Two patients (5.2%) required re-exploration for bleeding, but hemorrhaging was easily controlled. The mean postoperative blood loss via chest tubes was 1058 ± 416 mL and ventilation support was required for 3 ± 5 days. Two patients needed percutaneous endoscopic gastrostomy. There were no instances of paraplegia or left vocal cord paralysis. Liver and gastrointestinal function was preserved in all patients. There were no deep wound infections, and postoperative pain was controlled by oral analgesics (Table 3). Three patients required re-exploration for evacuation of a remaining intrapleural clot and bleeding. All patients required blood transfusion. The perioperative mean transfusion requirement was 6 units of packed red cells (Table 2) and all patients received fresh frozen plasma and platelet concentrates. All patients except the eight who died in hospital were discharged without nursing care after an average postoperative hospital stay of 35 days.

**Table 3:** Postoperative morbidity

Type of morbidity	No. of patients
Exploration for bleeding	2 (5%)
Acute myocardial infarction	1 (3%)
Low cardiac output	6 (16%)
Renal dysfunction requiring dialysis	3 (8%)
Gastrointestinal bleeding or ischemia	2 (5%)
Deep vein thrombosis or pulmonary embolus	0 (0%)
Pneumonia or atelectasis	4 (11%)
Wound infection	0 (0%)
Sepsis	1 (3%)
Paraplegia	0 (0%)
Vocal cord paralysis	0 (0%)
Temporary tracheostomy	2 (5%)
Percutaneous endoscopic gastrostomy	2 (5%)
Transient neurological dysfunction	
Delirium	2 (5%)
Convulsion	1 (3%)
Permanent neurological dysfunction	
Cerebral infarction	1 (3%)
Cerebral hemorrhage	0 (0%)

## Discussion

Surgical results for total arch replacement have recently been improving, and several reports now show less than 5% surgical mortality [9,10]. This success shifts the goal from reducing surgical mortality and morbidity and improving the surgical procedure to improving the quality of life after surgery. However, replacement of the thoracic aorta in stages may not be feasible when extensive aneurismal and dissection disease in the arch to the descending aorta makes staging technically challenging or when the presence of symptoms may be related to more than one diseased aortic segment [11].

We consider that the conventional two-stage approach has a number of disadvantages. It is used because a median sternotomy does not allow for adequate exposure of the descending thoracic aorta and distal arch. Surgeons are therefore forced to make a number of compromises, such as a second painful incision, dissection through dense adhesions from the previous procedure, application of an aortic clamp near the proximal anastomotic site with an increased risk of bleeding, or repeated hypothermic circulatory arrest and repeated anesthesia. The difficulties encountered at the distal suture line were addressed by introduction of the elephant trunk technique, which allowed for technically easier graft-to-graft anastomoses, thus facilitating the second stage of the operation. However, the interval between the stages remains a cause for concern, as the recent literature indicates a mortality of 10% during this period among patients on the waiting list [12,13]. The need for a second operation brings additional risks. For instance, staged replacement of the entire thoracic aorta is associated with a mortality rate of 7% to 16%, a neurologic sequelae rate of 9% to 60%, a respiratory insufficiency rate of 32%, and a renal failure rate of 10% per session [6]. For stage 2 alone, Safi and colleagues reported an additional incidence of pulmonary complications in 32%, renal insufficiency in 16%, cardiac complications in 29%, and encephalopathy in 6% of their patients [13]. Furthermore, the need for repeated general anesthesia, a second extracorporeal circulation period, and circulatory arrest has to be taken into consideration.

Posterolateral thoracotomy, which is a widely used surgical approach to arch combined with descending aortic disease, was not considered optimal for this study. Proximal aortic cross-clamping between the left carotid and left subclavian arteries was impossible because of a concomitant atherosclerotic arch aneurysm and aortic dissection, and clamping injury to the aorta would carry the risk of progressive aortic disease. In this situation, cardiac protection as well as brachiocephalic and axillary artery reconstruction would be very difficult. Open proximal anastomosis combined with deep hypothermic circulatory arrest might pose some complications for reconstructing arch branches, proximal anastomosis in the ascending aorta, establishment of antegrade perfusion for CPB, infusion of a cardioplegic solution, and de-airing/removal of debris in the ascending aorta. These complications could result in a higher incidence of postoperative stroke, difficulty in hemostasis, and inadequate cardiac protection.

Doss et al. used a clamshell incision for patients whose underlying pathology would conventionally require a staged repair via two separate incisions, or for whom technical difficulties were anticipated at distal arch anastomoses due to sclerosis or dissection. Single-stage replacement of the thoracic aorta with a clamshell incision has been previously used with an operative risk comparable with that of the two-stage approach [14]. Clamshell incision is particularly advantageous for reoperative procedures because it permits easier access to the mediastinal structures. With this procedure, bicaval cannulation eliminates the need for central pulmonary artery cannulation and makes the administration of retrograde brain perfusion possible without the use of separate percutaneous catheters. Furthermore, it allows clear exposure of the phrenic and recurrent laryngeal nerves, thus reducing the possibility of injury. Transverse thoracotomy is a single symmetric incision which yields good cosmetic results with only two incisions and a good quality of life. Although it causes more pain than a median sternotomy, it is certainly better tolerated than a posterolateral thoracotomy [15]. Women especially benefit from this incision, as the scar is partially hidden by the breasts while multiple chest scars and breast asymmetry are avoided. Our results are similar to the findings of Kouchoukos and colleagues, who reported a low mortality rate, low morbidity rate, and a low reoperation rate for bleeding with the clamshell incision [8]. Our in-hospital mortality rate of 5.0% for elective surgery shows that the approach alone does not necessarily explain the high rates of mortality described by other authors for this technique [15].

It has always been our aim to try and improve surgical techniques, especially cerebral protection, which remains a major concern in aortic arch surgery. As for cerebral protection, various methods such as antegrade, retrograde cerebral perfusion or deep hypothermic circulatory arrest have been assessed, and the results for each of these have been reported [18,19]. While arch vessels are being reconstructed, hypothermic circulatory arrest without cerebral perfusion is a simple technique which involves perfusion of vessels of the head via a separate graft that is later anastomosed to the aortic graft [20,21]. There is no embolic risk of atheromatous plaque dislodgement into the arch vessels at the time of cannulation, and both the back flow and the clampless method prevent air and debris from falling into the arch vessels [19]. This technique can be used without the need for complicated perfusion circuits, and provides a better operative field for the graft anastomosis site containing cervical vessels but it has certain disadvantages. Not using cerebral perfusion has the drawback of a limited safe period. We have therefore proposed that the time without cerebral perfusion should not exceed 40 minutes because non-physiological perfusion is used [22,23]. In addition, the relatively long lower body ischemic time may result in renal or hepatic dysfunction, poor recovery of gastrointestinal function or bleeding. As the body remains hypothermic until proximal anastomosis, internal organs are better preserved than under room temperature. Although the average circulatory arrest time of the lower part of the body was 97 minutes, which was rather long, we did not observe any correlation between the postoperative serum creatinine peak value and circulatory arrest time. However, hemofiltration or hemodialysis has to be transiently performed in patients with preoperative renal dysfunction. In such cases, we will soon shorten the circulatory arrest time of the lower part of the body by using distal aortic perfusion during arch reconstruction. This allows for lower body perfusion even during distal anastomosis of the descending aorta or graft-to-graft anastomosis. This technique can be expected to reduce the duration of lower body ischemia and may improve the recovery of renal, hepatic or gastrointestinal function [24,25]. We believe that a shorter period without cerebral perfusion is better for cerebral protection and neurological recovery after surgery. We therefore adopted the arch-first technique so as to obtain better neurological outcomes, shorter surgical recovery, and an enhanced quality of life even in the late postoperative phase.

The present study has several limitations. First, because the study was retrospective and not controlled, we could not compare our central arterial cannulation method with femoral arterial cannulation or standard ascending aortic cannulation as a control. Second, because the number of patients was limited, the various clinical parameters relating to the onset of brain injury could not be identified. Third, since this was a clinical study, there was a spectrum of aortic diseases, varying pathological changes in aortic disease and differences in aorta-graft anastomoses. These points should be re-examined in a future study. Moreover, we are planning to evaluate the results of other approaches or techniques such as intraoperative stent grafting for extended arch aortic disease

## Conclusion

We have presented our early clinical results of the use of a recently refined arch-first technique during circulatory arrest with cerebral perfusion. This technique is still being developed, but some refinements have already made a definite contribution to better clinical outcomes. The arch-first technique is clearly superior to the conventional distal-first technique in terms of surgical mortality and morbidity related to neurological outcomes. It also provides clinical results comparable to those for selective cerebral perfusion, and has the advantage that thrombo-embolism is less likely. We believe that our improvement of the surgical technique involved here may serve as a useful reference and contribute to the improvement of aortic surgery.

## Competing interests

The authors declare that they have no competing interests.

## Authors' contributions

Conceived and designed the experiments: HI, HS,

Analyzed the data: HI, TI

Wrote the manuscript: HI, HM

All authors read and approved the final manuscript.
